# The influence of decisional conflict on treatment decision in pelvic organ prolapse—data from the SHADE-POP trial

**DOI:** 10.1007/s00404-024-07723-8

**Published:** 2024-09-06

**Authors:** Larissa E. Drost, Rachel D. M. de Jong, Marjan Stegeman, Arie Franx, M. Caroline Vos

**Affiliations:** 1 Department of Obstetrics and Gynaecology, Elisabeth-Tweesteden Hospital, Tilburg, The Netherlands; 2 Department of Obstetrics and Gynaecology, Erasmus Medical Center, Rotterdam, The Netherlands

**Keywords:** Shared decision making, Decisional support, Patient participation, Urogynaecology, Pelvic organ prolapse

## Abstract

**Purpose:**

Women with symptomatic pelvic organ prolapse are facing the choice between several treatment options and a potentially difficult decision. The aim of this study was to examine the effect of decisional conflict, patient characteristics and other decision-related factors on treatment decision in women with pelvic organ prolapse.

**Methods:**

Data from the SHADE-POP trial were used. Women with symptomatic pelvic organ prolapse who visited their gynaecologist for (new) treatment options were included. In all participants, demographical characteristics and validated questionnaires concerning decisional conflict (DCS), shared decision making (SDM-Q-9), information provision (SCIP-B), anxiety and depression (HADS) and satisfaction with care (PSQ-18) were collected 2 weeks after the visit. Analyses were performed using univariate and multivariate linear and logistic regression analyses.

**Results:**

Ninety six women with pelvic organ prolapse facing a treatment decision were included. An increase in decisional conflict as experienced by patients was related to the choice of more conservative treatment, such as pelvic floor muscle training or pessary, instead of surgery (*p* = 0.02). Shared decision making, better information provision and satisfaction with care were related to lower levels of decisional conflict (*p* = 0.001).

**Conclusion:**

Decisional conflict in women with pelvic organ prolapse favours conservative treatment instead of surgery. Gaining knowledge on the effect of decisional conflict, patient characteristics and other decision-related factors on treatment decision in pelvic organ prolapse will be a step towards a better-guided treatment decision and better patient-reported outcomes for this group of patients. NL 55737.028.15, 30-10-2016.

## What does this study add to the clinical work

**Table:** 

This study will be a step towards a better-guided treatment decision for women with POP. This can result in increased treatment adherence and better patient-reported outcomes.	

## Introduction

When seeking treatment for a medical condition, patients can be confronted with treatment decisions. Treatment options may be considerably different looking at treatment effects, side effects and possible complications. This is also the case for women with symptomatic pelvic organ prolapse (POP), who are confronted with the choice between several treatment options for POP. They may consider the decision a difficult one [1–3].

Women with POP can experience a bulging feeling or vaginal pressure with or without associated symptoms such as urinary, defecatory or sexual dysfunction [4]. These symptoms can have major effects on the quality of life and can be a reason to consult a clinician for treatment [5, 6]. Besides expectant management, generally three treatment options for POP can be discussed with the patient; pelvic floor muscle training (PFMT), a pessary or surgery [1–3]. None of these treatment options has been proven to be superior. Each option has specific advantages and disadvantages and as the choice for a treatment option depends on many different factors, the decision should be made together with the patient [7]. This process is known as shared decision making (SDM) and tends to result in improved satisfaction and less decisional conflict [8].

To facilitate SDM, a decision aid (DA) for women with POP was developed and tested in the randomised controlled SHADE-POP trial. Participants already seemed to be satisfied with SDM and information provision and the DA did not influence this [9]. This raises the question to what extent patients with POP experience decisional conflict at all and how this affects their treatment decision. Decisional conflict is defined as personal uncertainty about which option to choose [10]. No literature is available on decisional conflict in this patient group or the effect of decisional conflict on treatment choice in related medical conditions.

Moreover, little research has been conducted on how treatment decision is affected by patient characteristics, such as educational level, age and anxiety, or other decision-related factors such as SDM and information provision. Also, very few studies report on how these aspects affect one another.

The aim of this study was to gain more insight in the decisional process of women with POP. The effect of decisional conflict on treatment decision was explored. Patient characteristics such as age, educational level, levels of anxiety and depression and other decision-related aspects such as satisfaction with SDM, satisfaction with information provision, satisfaction with care and DA use were also examined.

## Methods

### Study design

This study was a secondary analysis of data from the SHADE-POP trial. The SHADE-POP trial is a cluster randomised controlled trial exploring the effects of an online DA, considering different treatment options, on women with symptomatic POP [9]. Patients were included in the study between September 2017 and April 2021. Data from both trial arms were analysed together. The study was approved by the Medical Ethical Research Committee Brabant, Tilburg, The Netherlands (NW 2015–62). The trial was registered as the SHAred DEcision making in Pelvic Organ Prolapse (SHADE-POP) trial, NL 55737.028.15.

### Study population and data collection

Women with symptomatic POP who visited their gynaecologist for (new) treatment options were included. Inclusion criteria included eligibility for at least two treatment options and the ability to use the internet to fill out questionnaires. Exclusion criteria were a history of gynaecological cancer, no internet access, insufficient knowledge of the Dutch language, more than one previous prolapse surgery, prolapse surgery in the past 2 years or participation in another study concerning treatment of POP [11]. All patients signed written informed consent. Questionnaires were sent to the patients by e-mail at four moments in time. The baseline questionnaire, which was sent 2 weeks after the first consultation and before initial treatment was started, was used for the analyses in this paper. Follow-up questionnaires were sent at 6 months, 12 months and 24 months after baseline.

### Measures

Patients were requested to fill out several baseline characteristics, including self-reported educational level, parity and preferred treatment option. The decisional conflict scale (DCS) was used to evaluate decisional conflict concerning treatment choice [12, 13]. To evaluate the patients’ perceived level of involvement in SDM the shared decision-making questionnaire (SDM-Q-9) was used [14, 15]. Satisfaction with information provision was measured with the satisfaction with cancer information profile (SCIP-B) questionnaire and levels of anxiety and depression were determined using the hospital anxiety and depression scale (HADS) [16, 17]. Satisfaction with care was measured with the patient satisfaction questionnaire (PSQ-18) and health-related quality of life was evaluated by the EuroQol-5D, the Pelvic Floor Disability Index (PFDI-20), the pelvic floor impact questionnaire (PFIQ-7) and the POP/urinary incontinence sexual functioning questionnaire (PISQ) [18–22]. All questionnaires are validated and consist of Likert scales of four to six items.

### Outcome measures

The primary outcome of this analysis was the effect of decisional conflict on treatment decision. Other patient characteristics such as age, educational level, levels of anxiety and depression and decision-related factors such as level of SDM from the patient perspective, satisfaction with information provision, satisfaction with care and DA use were also examined.

### Statistical analysis

Statistical analysis was performed using IBM SPSS Statistics for Windows, version 28.0 (IBM, Armonk, NY, USA). Tests were two-sided and a *p* value of < 0.05 was considered statistically significant. Baseline patient characteristics were reported by means and standard deviations. Linear and logistic regression analyses were performed to analyse the relationship between the scores on the various questionnaires, educational level, DA use and treatment choice (conservative/surgical treatment). Factors were marked as confounders in case the regression coefficient changed > 10% after adding the factor to the regression analysis. To take into account the clustering at hospital level, multilevel linear regression analyses were performed to evaluate the effect of decisional conflict [23]. Two levels were identified: patients and hospitals. The model included the random intercept “hospital level” and the dependent variable “treatment choice”. No effect of clustering at hospital level was found. Therefore, only the results of the naïve analyses are mentioned in the results section.

## Results

**Fig. 1:**
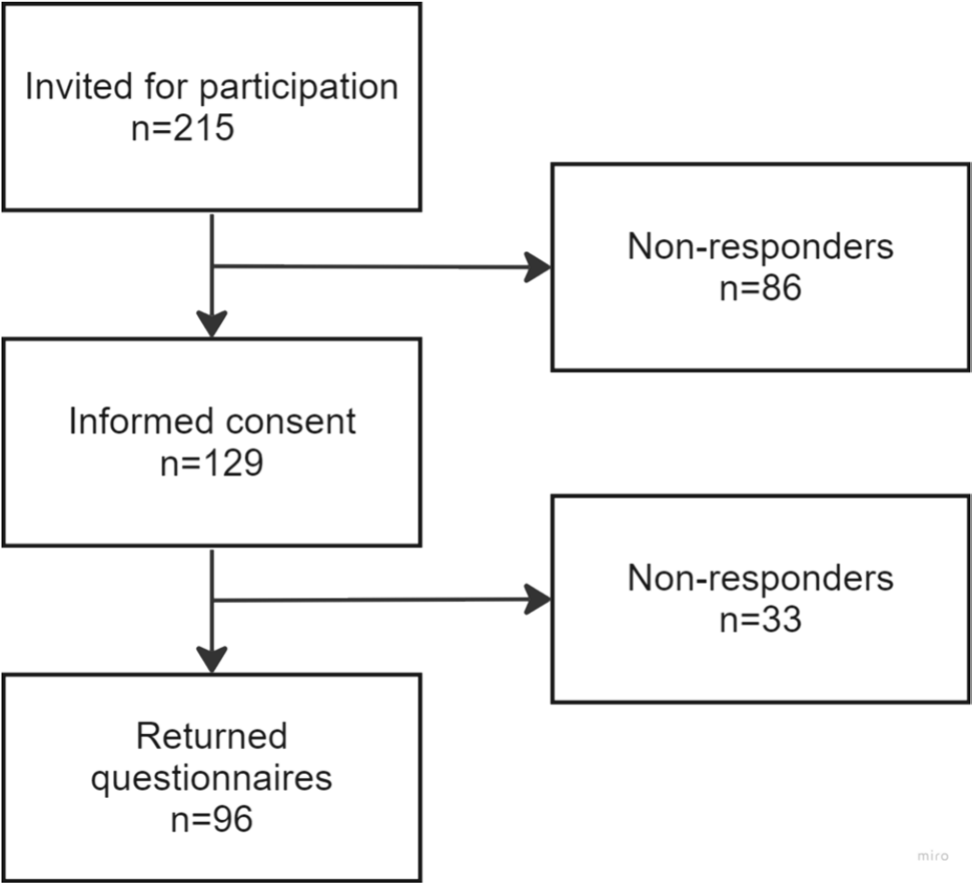
Flowchart enrolment numbers

Two hundred fifteen women who consulted a clinician for treatment of POP were eligible for inclusion in the study and received the patient information file. Subsequently, 129 patients signed the written informed consent form and were included in the study. 96 (44.7%) completed the first set of questionnaires. All patients to whom the DA was provided confirmed that they had used the DA. Figure 1 shows a flowchart of the study with enrolment numbers. The number of patients recruited per hospital varied between 7 and 60. One hospital switched from the usual care group to the DA group after the inclusion of 39 patients. Due to the COVID-19 pandemic, enrolment of patients was very challenging. The DA was made publically available after the pandemic. Therefore, continuation of the trial was not possible.

The mean age of the patients who completed the first set of questionnaires was 62.0 years. Table 1 shows baseline characteristics such as age, BMI, parity, educational level, DA use and preferred treatment option. In Table 2 the results of the questionnaires concerning decisional conflict, SDM, information provision, anxiety and depression and satisfaction with care can be seen. Severity of symptoms as measured by the PFDI-20, the PFIQ-7 and the Euroqol-5D and the impact on sexual functioning as measured by the PISQ-12 can also be seen in Table 2.

**Table 1:** Patient characteristics

	Completed questionnaires at T1 (*n* = 96)	Did not complete questionnaires at T1 (*n* = 33)	*p* value
Age in years, mean (SD)	62.0 (8.16)	63.8 (9.19)	0.30
BMI, mean (SD)	25.6 (3.50)		
Parity, mean (SD)	2.42 (1.22)		
Educational level, *n* (%)			
Low	17 (18%)		
Medium	51 (53%)		
High	28 (29%)		
Decision aid use, *n* (%)			0.94
Yes	40 (42%)	14 (42%)	
No	56 (58%)	19 (58%)	
Hospital, *n* (%)			
1	60 (63%)	17 (52%)	0.75
2	12 (13%)	7 (21%)	
3	7 (7%)	3 (9%)	
4	5 (5%)	2 (6%)	
5	12 (13%)	4 (12%)	
POP-Q stage, *n* (%)			0.43
1	6 (6%)	1 (3%)	
2	52 (54%)	22 (67%)	
3	38 (40%)	10 (30%)	
Preferred treatment option, *n* (%)			
Expectant management/PFMT	12 (13%)		
Pessary	47 (49%)		
Surgery	36 (38%)		
Other	1 (1%)

**Table 2:** Results of questionnaires

Outcome	Number of items	Range of scores	Total (*n* = 96), mean (SD)
DCS Decisional conflict	16	16–80	29.50 (9.96)
SDM-Q-9 Shared decision making	9	9–54	46.58 (7.99)
SCIP-B Information provision	10	10–50	45.93 (5.90)
HADS Anxiety and depression	14	0–42	5.67 (4.49)
PSQ-18 Satisfaction with care	18	18–90	70.25 (7.57)
PFDI-20 Pelvic floor disability	20	0–80	18.73 (11.32)
PFIQ-7 Pelvic floor impact	7	21–84	28.40 (7.44)
PISQ-12^a^ Impact on sexual functioning	12	12–60	47.50 (5.56)
Euroqol-5D Impact on daily activities	5	5–25	6.71 (2.05)

^a^56 respondents filled out this questionnaire

### Effect on treatment choice

Logistic regression analysis was performed to investigate the relationship between treatment choice and decisional conflict. The effect of the level of SDM, information provision, anxiety and depression, satisfaction with care, age, educational level and DA use on treatment choice was also explored. The univariate analysis of the association between those patient characteristics and decision-related factors, and treatment choice is presented in Table 3. For unknown reasons, one patient had not made a treatment choice at the moment of the baseline questionnaire.

**Table 3:** Univariate logistic regression analysis of factors affecting treatment choice

	B	S.E	Sig	Exp(B)	95% C.I. for EXP (B)	
					Lower	Upper
Decisional conflict	−0.058	0.025	0.024*	0.944	0.898	0.992
Shared decision making	0.031	0.030	0.299	1.031	0.973	1.093
Information provision	−0.024	0.035	0.505	0.977	0.911	1.047
Anxiety and depression	0.089	0.048	0.066	1.093	0.994	1.201
Satisfaction with care	−0.015	0.028	0.597	0.985	0.932	1.041
Age	−0.034	0.027	0.210	0.966	0.916	1.020
Educational level			0.272			
Lower vs. higher	1.034	0.641	0.107	2.812	0.800	9.882
Medium vs. higher	0.427	0.510	0.403	1.532	0.564	4.162
Lower vs. medium	0.607	0.567	0.284	1.836	0.605	5.572
Decision aid use	0.214	0.431	0.620	1.238	0.532	2.881

* indicates statistical significance

An increase in decisional conflict of the patient was significantly associated with the choice of a more conservative treatment option (*p* = 0.02). One point of increase on the DCS reduced the odds of choosing an invasive treatment option by 0.944. The decision for a specific treatment option was not affected significantly solely by the perceived level of SDM, information provision, anxiety and depression, satisfaction with care, age, educational level or DA use.

A multivariate logistic regression analysis was performed to predict the effect of all characteristics and factors together on treatment choice. The results are presented in Table 4.

**Table 4:** Multivariate logistic regression analysis of factors affecting treatment choice

	B	S.E	Sig	Exp(B)	95% C.I. for EXP (B)	
					Lower	Upper
Decisional conflict	−0.187	0.057	0.001*	0.829	0.741	0.928
Shared decision making	0.056	0.050	0.261	1.058	0.959	1.167
Information provision	−0.120	0.057	0.034*	0.887	0.793	0.991
Anxiety and depression	0.094	0.055	0.088	1.099	0.986	1.225
Satisfaction with care	−0.143	0.054	0.008*	0.867	0.780	0.964
Age	−0.007	0.033	0.839	0.993	0.930	1.060
Educational level			0.901			
Lower vs. higher	0.334	0.733	0.649	1.396	0.332	5.872
Medium vs. higher	0.123	0.589	0.834	1.131	0.357	3.586
Decision aid use	−0.791	0.529	0.135	0.453	0.161	1.278

* indicates statistical significance

In this analysis a significant effect of decisional conflict (*p* = 0.001), information provision (*p* = 0.034) and patient satisfaction with care (*p* = 0.008) on treatment choice was seen. One point of decrease on the DCS is related to a decrease of the odds of choosing an invasive treatment option of 0.829.

Information provision and satisfaction with care appeared to influence the relationship of decisional conflict and treatment choice. The perceived level of SDM, anxiety and depression, age, educational level and DA use did not have an influence on this relationship.

### Effect on decisional conflict

Furthermore, linear regression analyses were performed to investigate the effect of SDM, information provision, anxiety and depression, satisfaction with care, age, educational level and DA use on decisional conflict. Results are shown in Table 5. Simple linear regression analysis was used to test if SDM as experienced by the patient explained the perceived level of decisional conflict. The level of SDM explained 10.6% of the level of decisional conflict (F(1,94) = 11.193, *p* = 0.001). The level of information provision explained 17.8% of the level of decisional conflict [F(1,94) = 20.327), *p* =  < 0.001]. The level of patient satisfaction with care explained 46.7% of the level of decisional conflict [F(1,94) = 82.436, *p* =  < 0.001] and thus had the biggest effect. Levels of anxiety and depression, age, educational level and DA use did not have a significant effect on the level of decisional conflict.

**Table 5:** Univariate linear regression analysis of factors affecting decisional conflict

	*R*^2^	Unstandardised coefficients		Standardised coefficients	*t*	Sig	95% C.I. for B	
		B	S.E	B			Lower	Upper
Shared decision making	0.106	−0.407	−0.122	− 0.326	− 3.346	0.001*	−0.648	−0.165
Information provision	0.178	−0.712	0.158	−0.422	− 4.509	< 0.001*	−1.025	−0.398
Anxiety and depression	0.011	0.229	0.228	0.103	1.005	0.317	−0.223	0.681
Satisfaction with care	0.467	−0.899	0.099	−0.684	−9.079	< 0.001*	−1.096	−0.703
Age	0.026	0.203	0.129	0.160	1.572	0.119	−0.054	0.460
Educational level	0.009	1.360	1.507	0.093	0.902	0.369	−1.633	4.353
Decision aid use	0.000	−0.300	2.073	− 0.015	− 0.145	0.885	−4.415	3.815

* indicates statistical significance

## Discussion

This study aimed to gain insight in the decisional process of women with POP. In our study population, an increase in decisional conflict as experienced by the patient was associated with the choice of a more conservative treatment option. Other patient characteristics and decision-related factors were not associated with treatment choice.

It seems comprehensible that patients who experience more difficulty when choosing a treatment option opt for the more conservative alternative. After all, changing conservative treatment to a more invasive option is still possible and the other way around is not. However, we are the first to show this effect, as no data of the effect of decisional conflict on treatment choice are known from literature. This finding may be explained by the possible side effects of the treatment options. For PFMT, very few side effects have been described [24]. For pessary treatment, several side effects have been described that are often easy to treat and are reversible [1]. For more invasive treatment, more (irreversible) complications and adverse effects have been reported [3, 11].

This study showed no association of age with treatment choice. There is limited research on how age affects treatment choice. A study in oncological patients shows that young women are more likely to accept more drastic therapies than older women [25]. However, POP is a benign health problem and survival is not affected by treatment choice. Nevertheless, for older women comorbidities may be a reason to choose a conservative treatment option.

Furthermore, this study shows no effect of a DA on treatment choice. A Cochrane review on the effects of DAs shows that DAs reduce the number of people choosing invasive surgery in favour of more conservative options [26]. In our study, all patients who were offered the DA indicated that they used the DA, but it is unknown to what extent they used it. Moreover, our DA was not developed to affect treatment choice but to increase patient satisfaction with SDM and information provision.

The relation between patient characteristics, SDM, information provision and decisional conflict was also investigated. We found that the level of SDM is significantly correlated to decisional conflict. In studies of the choice for a mode of delivery or for a treatment for vestibular schwannoma an increase in SDM was associated with less decisional conflict [27, 28]. Also, information provision as measured by the SCIP-B questionnaire seems to be associated with decisional conflict in our population. This reconfirms several studies in oncological patients which show that adequate information provision decreases decisional conflict [29, 30].

In our data, confounding factors were information provision and patient satisfaction. In the univariate logistic regression analysis, no effect of these factors on treatment choice was found. However, the multivariate logistic regression showed an effect of information provision and patient satisfaction. This can be explained by the overlap in questions between the questionnaires used. The DCS for decisional conflict contains three questions which are closely related to the questions in the SCIP-B questionnaire for information provision; whether patients know which treatment options are available for their specific situation, whether they know what the advantages are and whether they know what the risks or side effects are of each option. Besides information provision, other questions in the DCS concern value clarity, support from others, uncertainty and satisfaction with decision making. This last question has an overlap with the PSQ for patient satisfaction. No collinearity of the questionnaires was found in the multivariate analysis.

One of the strengths of this study is the uniqueness of the data. We are the first to show the effect of decisional conflict on treatment decision in women with POP. It is known that many patients can experience decisional conflict, with gynaecology being one of the most commonly reported clinical decision contexts [31]. Women with pelvic floor disorders are known to experience decisional conflict as well, however this has not been studied for women with POP specifically [32].

A limitation of our study is its small sample size. As it concerns a secondary analysis of data from the SHADE-POP trial, a power calculation for the primary outcome of this study is not available. Unfortunately, the SHADE-POP trial itself did not reach power due to the COVID-19 pandemic. Furthermore, selection bias cannot be excluded with a 44% inclusion rate of eligible patients. Since questionnaires were only available on the internet, patients with limited computer skills may have refrained from participation. Data on health literacy were not collected. However, patients were asked for their educational level, which turned out to be relatively high. As a higher educational level is significantly correlated to better health literacy, educational level may have affected the decisional process [33, 34].

## Conclusion

Decisional conflict in women with POP is associated with the choice for a more conservative treatment instead of surgery. No effect of other patient characteristics or decision-related factors was found. If the different aspects of the decisional process can be improved, for example by a DA, we will be able to reduce decisional conflict in the future. Moreover, it will be interesting to see if patients adhere to their chosen treatment option in the future. In the SHADE-POP trial patients will be followed for 2 years, including a questionnaire on decisional regret, and patient files will be checked for treatment adherence. Our findings will be a step towards a better-guided treatment decision for women with POP which will result in increased treatment adherence and better patient-reported outcomes. 

## Data Availability

The data that support the findings of this study are available on request from the corresponding author, upon reasonable request.
